# The contribution of neighbours to an individual's risk of typhoid outcome

**DOI:** 10.1017/S0950268815000692

**Published:** 2015-05-04

**Authors:** D. L. CHAO, J. K. PARK, F. MARKS, R. L. OCHIAI, I. M. LONGINI, M. E. HALLORAN

**Affiliations:** 1 Vaccine and Infectious Disease Division, Fred Hutchinson Cancer Research Center, Seattle, Washington, USA; 2 The International Vaccine Institute, Seoul, South Korea; 3 Department of Biostatistics, College of Public Health and Health Professions, and Emerging Pathogens Institute, University of Florida, Gainesville, FL, USA; 4 Department of Biostatistics, School of Public Health, University of Washington, Seattle, Washington, USA

**Keywords:** *Salmonella* (Typhi), typhoid fever (*S*. Typhi), vaccination (immunization)

## Abstract

An individual's risk of infection from an infectious agent can depend on both the individual's own risk and protective factors and those of individuals in the same community. We hypothesize that an individual's exposure to an infectious agent is associated with the risks of infection of those living nearby, whether their risks are modified by pharmaceutical interventions or by other factors, because of the potential for transmission from them. For example, unvaccinated individuals living in a highly vaccinated community can benefit from indirect protection, or living near more children in a typhoid-endemic region (where children are at highest risk) might result in more exposure to typhoid. We tested this hypothesis using data from a cluster-randomized typhoid vaccine trial. We first estimated each individual's relative risk of confirmed typhoid outcome using their vaccination status and age. We defined a new covariate, *potential exposure*, to be the sum of the relative risks of all who live within 100 m of each person. We found that potential exposure was significantly associated with an individual's typhoid outcome, and adjusting for potential exposure affected estimates of vaccine efficacy. We suggest that it is useful and feasible to adjust for spatially heterogeneous distributions of individual-level risk factors, but further work is required to develop and test such approaches.

## INTRODUCTION

An individual's risk of infection can depend both on the individual's own risk and protective factors and those of individuals in same community. For example, it is well known that the direct effect of a vaccine can lower a vaccinee's susceptibility to infection, while widespread vaccination could confer indirect protection to both vaccinees and non-vaccinees by lowering disease prevalence [1–3]. Analogously, living in a low-risk community, perhaps in households with better access to clean water, could also lower an individual's risk of infection.

A person with a high risk of infection could have an increased probability of transmitting the pathogen to nearby individuals, which would increase their risks. We hypothesize that an individual's risk of exposure to a pathogen is associated with the risks of infection of people living nearby because of the potential for transmission from them. We test this hypothesis using data from a cluster-randomized typhoid vaccination trial. Typhoid fever, a result of infection with *Salmonella enterica* serovar Typhi, is responsible for an estimated 11·9 million cases per year [4]. In typhoid-endemic areas, young children are at higher risk of typhoid illness than adults [5–9], and high population density is also associated with increased typhoid risk [7, 9]. The Vi capsular polysaccharide vaccine has been shown to be moderately effective in reducing typhoid disease risk [10]. The primary analysis of the trial we analyse here found that unvaccinated individuals in vaccinated clusters had 44% lower incidence of confirmed typhoid illness compared to unvaccinated individuals in control clusters [11]. However, this estimate includes direct protection (biological protection of vaccinees) and indirect protection (reduced exposure to typhoid because of population-level vaccine coverage). Indirect protection appeared to extend beyond the boundaries of the study clusters – control clusters near vaccinated clusters also had low disease incidence [12]. We take this as evidence that an individual's risk of typhoid infection is associated with the risks of people living nearby.

**Fig. 1.**
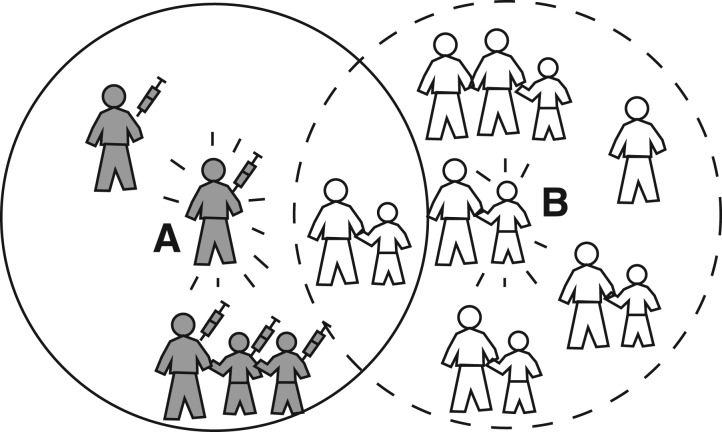
Illustration of how neighbours could affect an individual's risk of disease outcome. Here, adults and children are represented as large and small icons, respectively, and their positions represent the locations of their residences. Vaccinated individuals are shaded. The vaccinated adult indicated by ‘A’ may be exposed to typhoid by persons living nearby, shown within the solid circle. This individual may have a low risk of infection both because of the individual's own risk factors (adult age and vaccination status) and because of the relatively low risk of everyone nearby. The unvaccinated child indicated by ‘B’ is at high risk not only because of young age (a risk factor for typhoid outcome in this study) and lack of vaccination, but also because of the number of unvaccinated individuals and children living nearby. The unvaccinated adult and child in the middle of the diagram might contribute to the risk of both individuals ‘A’ and ‘B’. We define an individual's ‘potential exposure’ to an infectious agent to be the sum of the relative risks of those living nearby.

Here, we explore the effect of neighbours on an individual's risk of typhoid outcome in a typhoid vaccine trial. A pharmaceutical modifier of risk (vaccination) and a non-pharmaceutical modifier (age) were used to make an initial estimate of each individual's relative risk of typhoid outcome. We defined an additional covariate, *potential exposure*, to be the sum of the relative risks of all who live within 100 m of each person (Fig. 1). We explored the effects that adjusting for potential exposure had on estimates of vaccine efficacy in this trial.

## METHODS

### Typhoid vaccination trial data

**Fig. 2.**
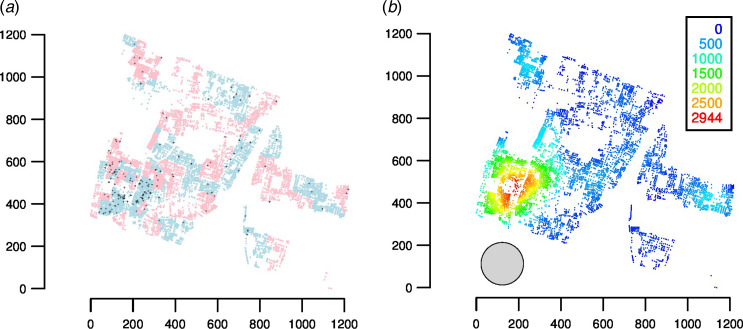
Maps of the study site and the potential-exposure measure. (*a*) Points in red are households in typhoid-vaccinated clusters, points in blue are in hepatitis A (control) vaccinated clusters. Units on the axes are in meters. Each typhoid case is plotted as a small + symbol. (*b*) Heat map of the potential-exposure measure of study participants. Each dot represents a trial participant's residence, with the colour based on the individual's potential exposure, as indicated by the key at the top right, based on the coefficients from model 3^(3)^ in Table 2. A grey circle with a 100-m radius is drawn in the lower left to indicate the spatial scale of the potential-exposure measure.

We re-analysed data from a cluster-randomized trial of Vi capsular polysaccharide typhoid vaccine that took place in an urban slum in Eastern Kolkata (ClinicalTrials.gov identifier NCT00125008), described in Sur *et al.* [11]. The institutional review boards of the International Vaccine Institute, the National Institute of Cholera and Enteric Diseases, and the Indian Council of Medical Research approved the study. Written consent was granted to use participant data in analyses. In brief, a population of 62 756 individuals ( 11 504 households) was divided into 80 geographical clusters. Clusters were assigned to be vaccinated with typhoid vaccine or a control vaccine (hepatitis A). Individuals aged ⩾2 years and not pregnant were eligible for vaccination. Of the 61 280 age-eligible individuals, 18 869 were vaccinated in the 40 typhoid-vaccinated clusters and 18 804 were vaccinated in the 40 control-vaccinated clusters. Vaccines were administered in late 2004. Surveillance in nearby clinics for febrile illness lasted to December 2006. Individuals presenting with fever for at least 3 days were seen by a study physician to diagnose typhoid by blood culture. The blood sample was obtained after informed consent. Locations of vaccinated and unvaccinated clusters and the residences of confirmed typhoid cases are mapped in Figure 2*a*.

### The potential-exposure measure

We define an individual's *potential exposure* to a pathogen based on the relative risks of infection of individuals living nearby. We assume a study of *N* individuals *i* on which *K* covariates *x*_*ik*_, *i* = 1, …, *N, k* = 1, …, *K*, have been observed. We assume a Cox proportional hazards regression model is used to obtain initial estimates of the contributions of the covariates to the outcome of interest. The hazard ratio estimates from the Cox model can be used as estimates of relative risk, and an initial estimate for an individual *i*'s relative risk of infection is:
1
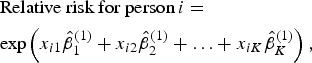


where 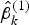 is the coefficient for covariate *k*, and the superscript ^(1)^ indicates it is the initial estimate. The relative risk is defined with respect to a reference group who are defined by certain values of *x*.

We define an individual's *potential exposure* to be the sum of everyone's relative risk of infection living within a certain distance of that individual. An initial estimate for an individual *i*'s *potential exposure* is:
2
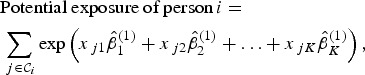


where *C*_*i*_ is the set of individuals within a designated distance of person *i* (excluding the person *i*) and 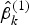 is the initial estimated coefficient for covariate *k* from equation (1). Although we define the contact set, *C*_*i*_, for each individual *i* as those living nearby, other proxies for closeness such as household or social network membership could be used. Figure 1 illustrates who contributes to the potential exposure of a person.

This potential-exposure estimate can be treated as a covariate that contributes to an individual's risk of infection. Each person *i* would therefore have a relative risk of infection of:3
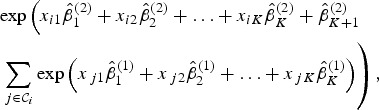


where 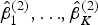 are newly estimated coefficients for covariates 1, …, *K* and 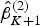 is the coefficient for the potential exposure. 
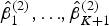
 can be estimated using a Cox proportional hazards regression.

Once the potential-exposure term is added to obtain equation (3), the coefficients 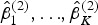 may differ from the coefficients 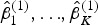 derived in equation (1). Therefore, these coefficients can be updated iteratively, estimating coefficients for iteration *m*, 
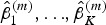
 using the coefficients from the previous iteration, *m* – 1, until the estimates converge.

Analyses were performed using R version 3.0.2 [13]. The Wilcoxon rank sum test was used to compare the prevalence of risk factors between typhoid cases and non-cases. The Cox proportional hazards regression model (coxph) from the Survival package [14, 15] was used with the Efron method for resolving ties [16]. Cluster assignment was taken into account in the analyses using the built-in ‘cluster’ option of coxph. Stepwise model selection by Akaike's Information Criterion (AIC) was performed using stepAIC from the MASS package [17].

## RESULTS

In the trial participants, an individual's typhoid vaccination status and age were associated with the risk of confirmed typhoid infection. The proportion vaccinated was lower in typhoid cases than non-cases, and the age of typhoid cases was significantly lower than that of non-cases (Table 1, *P* < 0·001). Using a Cox proportional hazards model regression with young unvaccinated children ( <5 years) as a reference group, older children (ages 5 to <15 years) had a risk of typhoid outcome that was 57% [95% confidence interval (CI) 39–81] of that of young children, and adults (those aged ≥15 years) had a risk that was 6% (95% CI 3–11) that of young children (Table 2, model 1). Typhoid vaccination was associated with a 64% (95% CI 42–77) lower risk of typhoid outcome (Table 2, model 1). This is an estimate of *total protection* of vaccination, which includes both direct and indirect protection from vaccination [18].

**Table 1.** Summary of characteristics of confirmed typhoid cases and non-cases

	Trial participants			Total population		
	Cases	Non-cases	*P*	Cases	Non-cases	*P*
*N*	130	37 543		177	62 579	
typhoid vaccination	34	18 835	<0·001	34	18 835	0·002
Age, years (mean)	10·4	28·3	<0·001	10·8	28·4	<0·001
% female	45·4%	47·5%	0·64	44·1%	46·2%	0·58
No. of people living within 100 m (mean)	6140	4284	<0·001	6244	4559	<0·001
% typhoid-vaccinated within 100 m (mean)	23·8%	30·2%	<0·001	24·3%	29·5%	<0·001
100 m potential exposure (mean)	1466	930	<0·001	2047	1393	<0·001

The first three columns summarize trial participants, who were vaccinated against typhoid or against hepatitis A (control vaccination). The last three columns summarize the total population of the study area, including those who did not participate in the trial. The Wilcoxon rank-sum test was used to obtain *P* values.

**Table 2.** Estimated vaccine effectiveness and relative risks for typhoid outcome

Term	V̂ (%)	95% CI	exp(β̂)	95% CI	*P*
Model 1					
Typhoid vaccination	63·7%	42·0 to 77·2	0·363	0·228–0·580	<0·0001
5–14·9 years			0·565	0·394–0·809	0·002
⩾15 years			0·059	0·033–0·108	<0·0001
Model 2a					
Typhoid vaccination	20·5%	−371·7 to 86·6	0·795	0·134–4·717	0·801
5–14·9 years			0·564	0·394–0·809	0·002
⩾15 years			0·060	0·033–0·109	<0·0001
Fraction vaccinated in cluster			0·274	0·016–4·675	0·371
Model 2b					
Typhoid vaccination	59·1%	32·0 to 75·3	0·409	0·247–0·680	<0·001
5–14·9 years			0·562	0·393–0·805	0·002
⩾15 years			0·060	0·033–0·108	<0·0001
Fraction vaccinated in 100 m			0·494	0·112–2·172	0·350
Model 2c					
Typhoid vaccination	56·9%	32·6 to 72·4	0·431	0·276–0·674	<0·001
5–14·9 years			0·565	0·395–0·807	0·002
⩾15 years			0·068	0·037–0·126	<0·0001
Number *unvaccinated* in 100 m/1000			1·139	1·053–1·231	0·001
Model 3^(1)^					
Typhoid vaccination	58·3%	34·8 to 73·3	0·416	0·266–0·650	0·0001
5–14·9 years			0·565	0·396–0·807	0·002
⩾15 years			0·069	0·037–0·127	<0·0001
100 m exposure/1000			1·488	1·172–1·890	0·001
Model 3^(2)^					
Typhoid vaccination	58·4%	35·0 to 73·4	0·416	0·266–0·650	0·0001
5–14·9 years			0·565	0·396–0·807	0·002
⩾15 years			0·069	0·037–0·127	<0·0001
100 m exposure/1000			1·471	1·166–1·856	0·001
Model 3^(3)^					
Typhoid vaccination	58·4%	35·0 to 73·4	0·416	0·266–0·650	0·0001
5–14·9 years			0·565	0·396–0·807	0·002
⩾15 years			0·069	0·037–0·127	<0·0001
100 m exposure/1000			1·471	1·166–1·856	0·001
Model 4^(3)^					
Typhoid vaccination	38·6%	6·5 to 59·7	0·614	0·403–0·935	0·023
5–14·9 years			0·813	0·606–1·091	0·168
⩾15 years			0·100	0·064–0·157	<0·0001
100 m exposure/1000			1·357	1·169–1·576	<0·0001

CI, Confidence interval.

Relative risks are reported as exp(estimate), with the coefficient estimated using a Cox proportional hazards model regression. Relative risks for models 1–3 were estimated for trial participants (typhoid and control vaccinees). V̂ in model 1 is the total vaccine effectiveness, which is simply (1 – exp()) × 100%. The fractions vaccinated in a cluster or within a 100-m radius are the fractions of total inhabitants vaccinated against typhoid. 100 m exposure/1000 is the potential exposure divided by 1000. Model 3^(1)^ computes potential exposure using the weight estimates from model 2, model 3^(2)^ computes potential exposure using the weight estimates from model 3^(1)^, and model 3^(3)^ computes potential exposure using the weight estimates from model 3^(2)^. Model 4^(3)^ is analogous to model 3^(3)^, except that the risks and potential exposure are estimated for everyone living in the study site, including non-participants.

We explored the association between an individual's risk of typhoid outcome and the vaccination coverage of people living nearby. The fraction vaccinated within an individual's study cluster was not significantly associated with typhoid outcome when an individual's age and vaccination status were taken into account (Table 2, model 2a). This is not surprising in this cluster-randomized study, where all typhoid vaccinees lived in typhoid-vaccinated clusters, which had an average of 61% coverage (range 38–79%). To mitigate the possible effects of cross-cluster contamination, we computed the vaccination coverage of people living with 100 m of each individual, regardless of cluster assignment. However, the vaccination coverage within 100 m of each individual was not significantly associated with typhoid risk (Table 2, model 2b). We found that the number of *unvaccinated* individuals living within 100 m of each individual was associated with typhoid risk, with a 1·14-fold higher risk for every 1000 unvaccinated people living within 100 m (*P* = 0·001; Table 2, model 2c).

We hypothesized that an individual's typhoid outcome is associated with the risks of those living nearby. If each unvaccinated child aged <5 years living nearby contributes to an individual's exposure to typhoid, each vaccinated child might contribute 64% less (based on the initial estimate of vaccine efficacy in Table 2, model 1), and a vaccinated adult might contribute (1·0−0·64) × 0·06 = 2·2% as much as an unvaccinated child. We define an individual's *potential exposure* to typhoid to be the sum of the relative risks of typhoid outcome of everyone living within 100 m of that individual, treating unvaccinated young children as the reference group. In this case, the potential exposure is based on the age and vaccination status of those living nearby, as illustrated in Figure 1. We assumed the contributions of non-study participants were the same as age-appropriate hepatitis vaccinees. A more complete definition of potential exposure is given in the Methods section.

This initial potential-exposure estimate was significantly associated with typhoid outcome (*P* = 0·001, Table 2, model 3^(1)^). Adjusting for potential exposure lowered the estimated effectiveness of typhoid vaccine from 64% to 58% (Table 2). The interpretation of the vaccine effectiveness estimate changes when adjusting for potential exposure. It is no longer purely a total effect as it takes into account interactions that cross study cluster boundaries. The estimates for the coefficients associated with a person's age and vaccination status changed when potential exposure was added to the model, so we recomputed each individual's potential exposure with the newly estimated coefficients and iterated until the exponentiated coefficients changed by <0·001, which occurred on the third iteration (Table 2, model 3^(3)^). The final vaccine effectiveness estimate was 58% (95% CI 0·35–0·73; Table 2, model 3^(3)^). The estimate of increased risk for each 1000-unit increase in potential exposure was 1·47 (95% CI 1·17–1·86). In other words, a study subject's risk of typhoid outcome was increased by a factor of 1·47 for each additional 1000 unvaccinated young children living within 100 m. The relative risk of those in the 3rd quartile *vs*. the 1st quartile of the potential-exposure measure was 1·73. Typhoid cases had higher potential exposure than non-cases (Table 1). A heat map of potential exposure of the trial participants is shown in Figure 2*b*. The confidence intervals of the coefficient values were estimated by the last iteration of the Cox proportional hazards model regression, and therefore do not take into account the additional variance that could be introduced (or removed) by iterating the estimation procedure. We re-estimated the potential-exposure coefficients (including the iteration steps) using 2000 bootstrap-resampled populations that preserved the sizes of the study clusters, and both the mean and the interval containing 95% of the vaccine effectiveness estimates from the resampled populations were nearly identical to those estimated by the last iteration of the Cox regression using the original data.

Potential exposure is correlated with population density and local vaccination coverage but appeared to be a better predictor of typhoid risk. The terms in the final model selected using a stepwise model selection by AIC were typhoid vaccination status, age, and potential exposure, while the fraction vaccinated against typhoid within an individual's cluster or within 100 m and the number of individuals living within 100 m were not in the final model.

For an alternative estimate of potential exposure, we estimated the relative risk of typhoid outcome for everyone living in the study area, including non-participants. The observed incidence of typhoid was substantially lower in non-participants. Reported incidence in hepatitis A (control) vaccinees was 0·73/100 000 person-days and in non-participants 0·35/100 000 person-days [11]. Thus, the estimated vaccine effect was lower when the entire population was included (Table 2, model 4^(3)^). The increased risk for each 1000-unit increase in potential exposure was 1·36 (95% CI 1·1–1·58).

## DISCUSSION

In the typhoid vaccination trial analysed here, an individual's risk of typhoid outcome was associated with the risks of people living nearby. We defined a new covariate, the *potential exposure*, i.e. the sum of the relative risks of individuals living nearby, as a proxy for the contribution of neighbours to an individual's risk of disease outcome. An individual's potential exposure was significantly associated with typhoid outcome, and appeared to be a better predictor of risk than population density and local vaccination coverage. We then estimated typhoid vaccine effectiveness adjusting for potential exposure to take into account the spatially heterogeneous distributions of typhoid vaccination status, age, and population density, three known major risk factors. Interestingly, increased age is associated with a greater reduction of typhoid risk than vaccination, so in theory those living in an area with fewer children could be at less risk of typhoid exposure than those living in highly vaccinated areas.

For simplicity, we assumed that everyone living within 100 m contributed equally to an individual's risk of infection. We explored other distance cut-offs and found that using substantially larger or smaller distances did not yield useful results (results not shown). The appropriate distance for inclusion in the potential-exposure measure probably depends upon the epidemiology of disease, local demography, and the study design. One could weight the contribution of neighbours by distance or assign higher weights to an individual's family members and other close contacts. One may expect population density to be associated with the risk of contracting enteric diseases, since crowding could lead to more transmission via an environmental route (e.g. contamination of water or latrines). For diseases with different modes of transmission, such as aerosol or sexual contact, the distance between residences could be a poor proxy for the connectedness between individuals. As an alternative, one could use social network information to inform a distance metric, or, for environmentally transmitted diseases, the interaction between individuals may be mediated by their distances to certain bodies of water rather than distances to each other [19]. The potential-exposure approach differs from the local efficacy measure of Emch *et al*. [20], which allows the protective efficacy of a vaccine to vary in space due to ecological differences and/or spatial variation in vaccination coverage. The potential-exposure approach estimates a single protective efficacy for vaccination and adjusts for the potential ‘exposure’ from nearby individuals.

The potential-exposure approach could be applied to data from cluster-randomized, individually randomized, or observational studies. In cluster-randomized studies, such as the one evaluated here, individuals may live near cluster boundaries, so unmeasured contamination across clusters may be an issue when estimating the effects of vaccination. A limitation of the approach is that it requires knowledge of the geographical location of the underlying population, although such information is increasingly being gathered by vaccine studies. In the present study, the estimates of potential exposure of those living on the edges of the study site are subject to edge effects because we did not have census data for those outside the geographical boundaries of the site, as can be seen in Figure 2*b* as lower apparent potential exposure in those living near the edges of the site.

We took two approaches to estimating potential exposure. In the first, we used the regression coefficients for individuals vaccinated with hepatitis A vaccine (control vaccine) to compute the contribution of the non-participants to potential exposure. This assumes that non-participants in both the vaccinated and control clusters had similar relative risks of confirmed infection as the control vaccinees, which is not supported by the data. The incidence in non-participants was considerably lower than in the participants, evidence of selection bias, which might be due to lower health-seeking behaviour or higher healthiness in non-participants. In the second approach, the trial non-participants were treated like control vaccinees to estimate coefficients. This also assumes the groups have similar risk. A third approach might be to include an indicator variable for being a non-participant. However, the two approaches we used in our analyses essentially bound the estimates that might be obtained through that analysis.

Adjusting for possible exposure to infectious agents from nearby individuals could improve estimates of intervention effects and risk of disease outcome. The potential-exposure measure proposed here is an initial attempt to create a proxy for exposure from neighbours, and more methodologically sound approaches could be developed. For example, including an individual's covariates and outcome in the estimation of his/her own potential exposure could introduce cycles that would hamper the convergence of coefficient estimates using an iterative estimation procedure. One could could instead estimate the potential-exposure coefficient values for each individual using only information from other individuals, in essence a jackknife of the data for each individual in the study population. However, for a dataset as large as the one used in the present study, this computationally expensive step would probably not alter the estimates. One could also use separate covariates to represent the contribution of each risk factor of neighbours rather than combining them in a single potential-exposure estimate. This would break the potentially problematic dependence of the estimates of the individual-level risk factor coefficients and the covariate(s) representing the contribution from neighbours. We defined potential exposure to be the sum of the relative risks of neighbours, but a more formal approach could define estimands for the contribution of a neighbour to an individual's risk, how to combine the contributions from multiple neighbours, and the estimand's relationship with direct and indirect effects from vaccination and other risk and protective factors. Further research is needed to elucidate how individuals contribute to the risks of others, how to use this knowledge to obtain more precise estimates of intervention effects, and how to leverage this information to improve the effectiveness of public health interventions.
